# Clinical Outcomes of the Adapted AAP 2019 Guidelines on Early Onset Sepsis in Thailand

**DOI:** 10.3390/antibiotics14101048

**Published:** 2025-10-20

**Authors:** Kanokwan Aeimcharnbanchong, Patraporn Jangmeonwai

**Affiliations:** Department of Pediatrics, Panyananthaphikkhu Chonprathan Medical Center, Srinakharinwirot University, Nonthaburi 11120, Thailand; phattaraporn@g.swu.ac.th

**Keywords:** Early Onset Sepsis Calculator, enhanced observation, antibiotic stewardship

## Abstract

**Background**: Antibiotic overuse in early-onset sepsis (EOS) remains a significant clinical challenge. Panyananthaphikkhu Chonprathan Medical Center adapted the 2019 American Academy of Pediatrics (AAP) guidelines by integrating the EOS calculator with enhanced observation. This study aimed to evaluate clinical outcomes before and after implementation in Thailand, focusing on timely initiation of empirical antibiotics in neonates with EOS and the reduction in unnecessary investigations and antibiotic exposure. **Methods**: This retrospective cohort observational study included neonates ≥ 35 weeks’ gestation. Participants were divided into two groups: “before” (1 February 2017–31 January 2018) and “after” (1 February 2023–31 January 2024) guideline implementation. Data were analyzed using Pearson chi-square, Mann–Whitney U-test, and binary logistic regression, with statistical significance defined as *p* < 0.05. **Results**: Among 3040 neonates (1639 before and 1401 after guideline implementation), antibiotic use declined from 11% to 7.9% (*p* < 0.001), with an Odds Ratio of 1.46 (95% Confidence Interval 1.14–1.87). Following the implementation of the Adapted AAP 2019 guidelines, a neonate with GBS septicemia was identified at birth with respiratory distress and was promptly started on antibiotics per the guideline. **Conclusions**: The Adapted AAP 2019 guidelines improved EOS management by reducing unnecessary investigations and antibiotic use while ensuring timely empirical antibiotic administration, as shown by the prompt management of a GBS septicemia case. Key to this reduction lies in the Adapted AAP 2019 guidelines, which provide clear definitions of EOS and recommend serial clinical observation for asymptomatic neonates born to mothers with risk factors for EOS.

## 1. Introduction

Neonatal early onset sepsis (EOS) is defined as blood or cerebrospinal fluid (CSF) bacterial infection of the newborn within 72 h after birth. The most common organism causing EOS is *Streptococcus agalactiae* (GBS) [1]. Most neonates with GBS EOS present clinically at or shortly after birth [2,3,4]. Approximately 80–90% of neonates with GBS EOS will be symptomatic within the first 48 h [3,5]. In a previous study by Michael, it was shown that under the CDC 2010 guideline, the percentage of neonates being treated with antibiotics is approximately 200 times higher than the actual incidence of EOS [6]. This overuse of antibiotics is primarily due to the difficulties in diagnosing EOS, which arise from nonspecific clinical signs and the low specificity of laboratory tests [7]. Moreover, the incidence rate of EOS has declined significantly—from 1.8 cases per 1000 live births in 1990 to 0.23 cases per 1000 live births in 2015—largely due to the implementation of universal maternal antenatal screening and GBS intrapartum antibiotic prophylaxis (IAP) [4].

Antibiotic overuse in early life can disrupt the developing gut microbiota, which is essential for immune maturation, digestion, and protection against infections [8]. Commensal aerobic and anaerobic bacteria play a critical role in colonization resistance, preventing the establishment of pathogenic and antibiotic-resistant organisms [9]. Disruption of this balance results in bacterial dysbiosis, which increases the risk of early complications such as necrotizing enterocolitis and fungal infections [10,11]. Dysbiosis has also been associated with long-term adverse outcomes, including allergies, obesity, diabetes, and inflammatory bowel disease [12]. Excessive use of antibiotics, particularly broad-spectrum agents, further promotes the emergence of resistant bacteria and reduces the gut’s protective capacity against pathogens [13]. Beyond biological effects, antibiotic overuse contributes to higher rates of intensive care unit admission, increased healthcare costs, and unnecessary separation of mother and neonate [14].

At Panyananthaphikkhu Chonprathan Medical Center, the incidence rate of culture-proven EOS in late preterm and term neonates is 0.22 per 1000 live births [15], mirroring the global trend of decreasing EOS cases. Nonetheless, the management of EOS remains challenging, particularly in balancing timely antibiotic use with minimizing unnecessary interventions. In 2019, the American Academy of Pediatrics (AAP) introduced a clinical report outlining three primary approaches for EOS risk assessment in infants born at ≥35 weeks’ gestation: (1). categorical risk assessment, (2). neonatal Early Onset Sepsis Calculator (EOSC), and (3). enhanced observation, each presenting distinct limitations [16]. The categorical risk assessment, which recommends a full sepsis evaluation for all infants with maternal fever, often leads to unnecessary laboratory testing and empirical antibiotic use in well-appearing neonates. To handle this, the EOSC was developed, which assesses EOS risk based on several factors, including the incidence of EOS, gestational age, maternal antepartum temperature, duration of rupture of membranes (ROM), maternal GBS status, and type of IAP, as well as neonatal clinical status at birth. The EOSC stratifies infants into three risk levels with corresponding management recommendations: (1). routine neonatal care, (2). obtaining a blood culture and monitoring vital signs for at least 24 h, or (3). starting empirical antibiotic therapy after obtaining a blood culture [17,18,19,20,21,22,23,24,25]. Although the use of the EOSC has been shown to reduce the use of empirical antibiotics, a previous retrospective study found that some neonates with culture-proven EOS might have been missed [26,27,28]. The enhanced observation approach focuses on treating only infants with clinical signs of illness and relies on serial physical examinations for well-appearing infants with maternal risk factors. While this strategy has demonstrated a reduction in antibiotic use, concerns remain that some cases of proven sepsis could still be missed [29,30,31,32].

At Panyananthaphikkhu Chonprathan Medical Center, we have adapted the AAP 2019 guidelines by implementing the EOSC along with an enhanced observation protocol. These adaptations were introduced in 2023. This study aims to compare clinical outcomes before and after the implementation, with a focus on two main objectives: (1). ensuring timely administration of empirical antibiotics to neonates with EOS, and (2). reducing the use of laboratory investigations and unnecessary empirical antibiotics. By analyzing these outcomes, we aim to assess the effectiveness of this approach in optimizing EOS management while minimizing overtreatment in neonates.

## 2. Results

**Figure 1:**
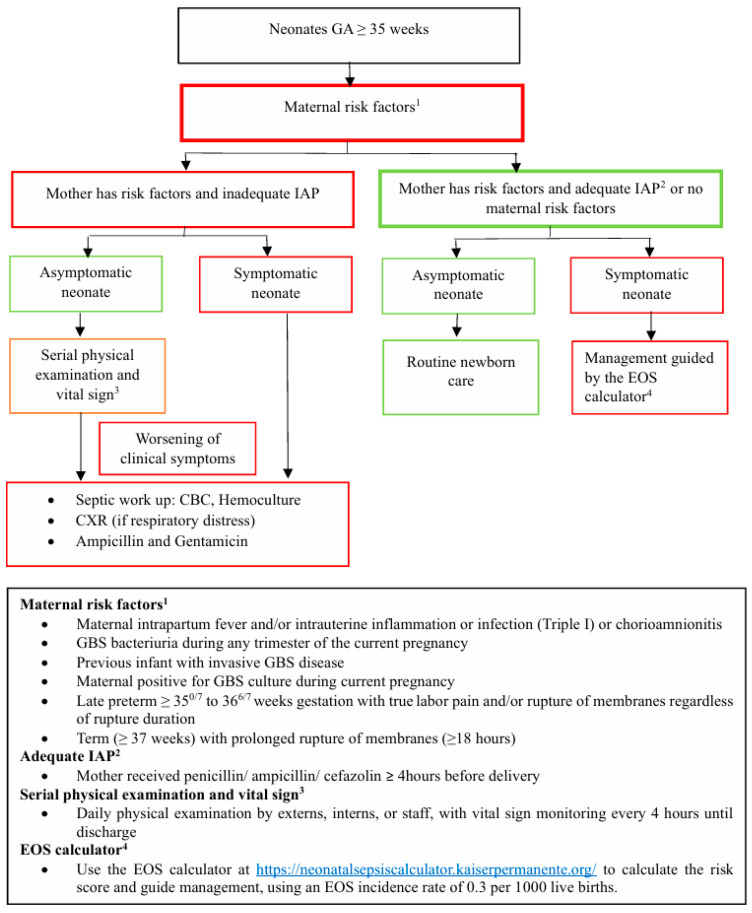
The adapted AAP 2019 guidelines.

In this study, 3040 neonates were enrolled, with 1639 born before and 1401 born after the implementation of the adapted AAP 2019 guidelines (Figure 1). As shown in Table 1, there were no significant differences in demographic data between the two groups. The mean gestational age, mode of delivery, birth body weight, and the proportion of male infants were all similar. Regarding maternal risk factors for EOS, there were 85 neonates (5%) before the implementation of the adapted AAP 2019 guidelines and 82 neonates (6%) after their implementation. The most common maternal risk factors in both periods were preterm labor and preterm ROM.

Culture-proven EOS remained rare in both periods. Before the implementation of the adapted AAP 2019 guidelines, one neonate with *Staphylococcus hominis* septicemia was identified. This 40-week gestation male infant presented with mucous bloody diarrhea, and his mother’s GBS status was unknown. He was successfully discharged after treatment. After implementing the adapted AAP 2019 guidelines, a 37-week gestation female neonate with GBS septicemia was identified. The only maternal risk factor was the unknown GBS status. At birth, she presented with respiratory distress and was placed on continuous positive airway pressure. She was promptly started on ampicillin and gentamicin for clinical illness, following the workflow outlined in the adapted AAP 2019 guidelines (Figure 1). She was successfully discharged after treatment.

Table 2 shows that before implementing the adapted AAP 2019 guidelines, clinical symptoms of illness were present in 17.8% (291) of neonates, which slightly decreased to 15% (210) after their implementation. The three most common clinical manifestations were respiratory distress (89% before vs. 91% after), feeding intolerance (4.5% before vs. 4.3% after), and hypotension (3.8% before vs. 4.8% after). No statistically significant differences were found in any of the clinical manifestations before and after the implementation of the Adapted AAP 2019 guidelines.

Table 3 shows that following implementation of the adapted AAP 2019 guidelines, the rate of investigations declined from 2.5% to 0.1% (*p* < 0.001), with the odds of investigations being significantly higher before guideline implementation (OR 35.92, 95% CI 4.93–261.47). Similarly, antibiotic use decreased from 11% to 7.9% (*p* < 0.001), with the odds of antibiotic use also being significantly higher before guideline implementation (OR 1.46, 95% CI 1.14–1.87).

## 3. Discussion

We studied 3040 neonates, including 1639 before and 1401 after implementation of the adapted AAP 2019 guidelines, to evaluate their clinical impact at Panyananthaphikkhu Chonprathan Medical Center. Implementation of the guidelines was associated with a substantial reduction in unnecessary investigations and empirical antibiotic use, while ensuring timely administration of antibiotics for neonates with culture-proven EOS.

These findings align with Cavigioli, F.’s study, which showed that the combined strategy of the EOSC and enhanced observation significantly reduced laboratory tests and antibiotic treatments in term and near-term newborns, without affecting EOS rates or mortality [33]. However, our study differs in the specific guidelines implemented. While both studies utilize the EOSC and enhanced observation, Cavigioli’s study applied the EOSC to all neonates born at ≥35 weeks gestational age, using enhanced observation only for those without maternal or perinatal EOS risk factors. In contrast, our adapted AAP 2019 guidelines begin by assessing maternal risk factors. If risk factors are present, enhanced observation is prioritized, leading to immediate investigation and antibiotic treatment if symptoms arise. If there are no maternal risk factors, or if adequate IAP were administered, we apply the EOSC for this group of neonates.

The adapted AAP 2019 guidelines can reduce investigations and antibiotic use by enhancing sepsis risk assessment and monitoring. The EOSC stratifies neonates into risk categories using clinical and laboratory data, guiding targeted investigations and more judicious antibiotic use. Enhanced observation provides ongoing monitoring to detect changes and avoid unnecessary treatments for stable neonates. Combining both methods offers a comprehensive approach to risk assessment and clinical decision-making. In contrast, before their implementation, the management of neonates with suspected EOS was guided by the CDC 2010 and AAP 2012 guidelines, along with physicians’ discretion and experience. This often led to extensive investigations and broader antibiotic use, as the guidelines recommended both investigation and antibiotic treatment for neonates showing signs of sepsis and for asymptomatic neonates born to mothers with chorioamnionitis, including late preterm neonates with EOS risk factors and inadequate IAP. They also advised investigating asymptomatic neonates with maternal EOS risk factors and inadequate IAP [33,34].

The most common clinical manifestation of illness in our study was respiratory distress, which aligns with findings from previous studies [34,35]. Before the implementation of the adapted AAP 2019 guidelines, neonates presenting with mild tachypnea lasting more than 4 h were routinely investigated. If a complete blood count showed leukocytosis, empirical antibiotics were often initiated. However, most of these neonates were later diagnosed with transient tachypnea of the newborn, reflecting a tendency toward over-investigation and antibiotic overuse. After the implementation of the adapted AAP 2019 guidelines, neonates in this group without maternal risk factors for EOS would receive routine vital sign monitoring without unnecessary interventions.

In our study, we found that 5% of neonates presented with maternal EOS risk factors before the implementation of the adapted AAP 2019 guidelines, and 6% of neonates presented with these risk factors after the implementation. This result is lower compared to previous studies, which reported incidences of 11% [22], 16.8% [23], and 23.4% [25] for neonates with maternal risk factors for EOS. Our study shows a lower incidence of EOS risk factors in mothers, possibly due to Thailand’s lack of routine GBS screening with vaginal swabs. The rate of vaginal swab culture for GBS was about 1% before and after implementing the adapted AAP 2019 guidelines, with a slight increase to 1.6% post-implementation. This limited screening may contribute to the observed lower maternal risk factors.

In this study, the incidence of culture-proven EOS was 0.6 per 1000 live births before and 0.7 per 1000 live births after the implementation of the Adapted AAP 2019 guidelines. This rate is higher than that reported in a previous 5-year study by Aeimcharnbanjong at Panyananthaphikkhu Chonprathan Medical Center during the 2016–2021 period [15]. The increased incidence observed in our study is likely due to the limited data collection period of only one year for each phase, both before and after the implementation of the Adapted AAP 2019 guidelines. This brief timeframe may not adequately represent the entire population.

In our hospital, We chose to integrate both EOS and enhanced observation approaches because we identified several limitations: (1). Prior retrospective studies have indicated that some neonates with culture-proven EOS might be missed when using either the EOSC [26,27,28] or the enhanced observation guideline alone [29,30,31,32]; (2). the EOSC does not recommend investigation and antibiotic therapy for symptoms like feeding intolerance and altered consciousness, which can also indicate possible EOS; and (3). Using empirical antibiotics for all neonates classified as having ‘clinical illness’ by the EOSC could contribute to antibiotic overuse. For example, neonates with conditions like transient mild pulmonary hypertension or transient tachypnea of the newborn may present with desaturation or rapid breathing that requires interventions such as oxygen, heated humidified high-flow nasal cannula or continuous positive airway pressure. These symptoms could be misclassified as clinical illness, leading to unnecessary antibiotic treatment.

The strengths of our study, which distinguish it from previous research, include the evaluation of the adapted 2019 AAP guidelines through dual-strategy integration and the use of a locally derived, population-specific incidence rate of early-onset sepsis (0.3 per 1000 live births), as reported by Aeimcharnbanjong [15]. Tailoring the EOSC’s incidence rate to the specific population at Panyananthaphikkhu Chonprathan Medical Center enhances both the accuracy and the relevance of our findings.

However, our study has several limitations. As a retrospective analysis, adherence to the implemented guidelines by individual physicians could not be fully verified, and data collection relied on medical records, which may have been incomplete. Conducted at a single center, the findings may not be generalizable to other settings with different populations or clinical practices. Additionally, the lack of routine GBS screening in Thailand may have resulted in fewer identified maternal EOS risk factors, potentially affecting baseline risk assessments and effectiveness of the EOSC.

Future multi-center, prospective studies are needed to validate these management strategies, particularly in Thailand and other low- and middle-income countries (LMICs) with differing routine GBS screening practices. Such implementation studies will provide critical insights into the feasibility, effectiveness, and safety of the guidelines across diverse healthcare settings.

## 4. Materials and Methods

### 4.1. Methods Study Design and Data Collection

This retrospective cohort observational study was conducted at Panyananthaphikkhu Chonprathan Medical Center, a tertiary care hospital and a center of excellence for neonates in an urban area of Thailand. The study was approved by the Committee on Human Rights Related to Research Involving Human Subjects at Panyananthaphikkhu Chonprathan Medical Center, Srinakharinwirot University, under ethical approval code 4.2/2567. We conducted the study following the Declaration of Helsinki. We did not require the patient’s informed consent as the data were anonymized. To ensure the confidentiality of the data from the participants’ records, we did not record any personal identifiers on the data collection sheet, and the data from participant records were not available to anyone except the researchers.

Inclusion and Exclusion Criteria

We included all neonates with a gestational age of ≥35 weeks, admitted during two distinct study periods:

1. Pre–adapted AAP 2019 guidelines period (1 February 2017–31 January 2018): This period was specifically chosen to ensure that no neonates received treatment according to the AAP 2019 guidelines. Neonates with suspected EOS were managed according to the CDC 2010 guidelines [36] and the AAP 2012 guidelines [37], supplemented by the clinical judgment of attending physicians. Suspected EOS was not defined by standardized criteria; inclusion was based on the treating physician’s assessment of clinical signs. EOS cases were classified as culture-proven EOS.
2. Post–adapted AAP 2019 guidelines period (1 February 2023–31 January 2024): This period was selected because the adapted AAP 2019 guidelines were formally implemented as the standard protocol at Panyananthaphikkhu Chonprathan Medical Center in January 2023. Prior to implementation, the neonatology team provided detailed guidance to all clinicians involved in neonatal care, including pediatricians and interns. During this period, periodic reviews of guideline implementation were conducted, and medical records consistently documented neonatal signs, diagnostic steps, and management. This system ensured that neonates were treated in accordance with the adapted AAP 2019 guidelines. Suspected EOS during this period was defined according to the EOSC guideline, and EOS cases were classified as culture-proven EOS.

Between 2019 and 2022, Panyananthaphikkhu Chonprathan Medical Center did not have a standardized protocol for EOS management; attending physicians used either the CDC 2010, AAP 2012, or AAP 2019 guidelines based on individual clinical judgment.

We excluded neonates with congenital multiple anomalies or those transferred for further treatment within the first 7 days, such as infants with moderate to severe hypoxia at birth and infants with severe congenital heart disease.

We reviewed medical charts and collected data. Then, we obtained data from medical charts such as gestational age, birth weight, sex, delivery type, maternal risk factors of EOS (maternal chorioamnionitis, previous infant with invasive GBS disease, GBS bacteriuria or maternal positive for GBS culture during current pregnancy, late preterm between 350/7 and 366/7 weeks’ gestation with true labor pain and/or ROM regardless of rupture duration, and term with ROM ≥ 18 h), neonatal clinical manifestations, neonatal laboratory, antibiotics and investigation which neonate received.

### 4.2. EOS Guidelines Before the Adapted AAP 2019 Implementation

The management of neonates with suspected infections primarily depended on physicians’ clinical judgment and was influenced by EOS recommendations derived from the CDC 2010 and AAP 2012 guidelines.

### 4.3. EOS Guidelines After the Adapted AAP 2019 Implementation

We integrated the EOSC and prioritize enhanced observation in the adapted AAP 2019 guidelines for the effective management of EOS in neonates over 35 weeks’ gestation, as shown in Figure 1.

The guidelines begin with an assessment of maternal risk factors. If maternal risk factors are present, IAP was inadequate, and the neonate shows signs of EOS, immediate investigation and antibiotic treatment are initiated. However, if the neonate shows no symptoms, enhanced observation is prioritized, with immediate investigation and antibiotic treatment initiated if symptoms develop.

For neonates whose mothers do not have risk factors for EOS, or if the mother has risk factors but received adequate IAP, the EOSC is applied to this group of neonates. The EOSC provides a clear definition of the clinical signs of EOS and the recommended treatment.

Key differences before and after the adapted AAP 2019 guidelines include: (1). the Adapted AAP 2019 guidelines providing explicit definitions of EOS, in contrast to the previous guidelines, which did not define EOS, and (2). the adapted AAP 2019 recommending serial clinical observation for asymptomatic neonates born to mothers with EOS risk factors who received inadequate IAP, whereas the previous guidelines recommended investigation and/or antibiotic treatment.

### 4.4. Definition

Feeding intolerance is defined as difficulty digesting enteral feedings, accompanied by increased gastric residuals, abdominal distension, and/or vomiting [38].

Hypotension is defined as a value below the 5th percentile for gestational and postnatal age [39].

Temperature instability is defined as a body temperature below 36.5 degrees Celsius or above 37.5 degrees Celsius [40].

Birth asphyxia is defined as an Apgar score at 5 min of less than 5 [4].

### 4.5. Statistical Analysis

Statistical analyses were conducted using IBM SPSS Statistics for Windows version 18 (IBM Corp., Armonk, NY, USA). The Pearson chi-square test or the likelihood chi-square test was used to compare proportions among categorical variables between two independent groups. The Mann–Whitney U-test was employed to compare differences between two independent groups when the dependent variables were ordinal or continuous but not normally distributed. The *p*-value for the odds ratios (OR) related to clinical outcomes before and after the implementation of the Adapted AAP 2019 guidelines was calculated using the Wald test in univariable binary logistic regression models. Statistical significance was identified using a two-sided *p* value less than 0.05.

## 5. Conclusions

The adapted AAP 2019 guidelines improved EOS management by reducing unnecessary investigations and antibiotic use while ensuring timely empirical antibiotic administration, as shown by the prompt management of a GBS septicemia case. Key to this reduction is the introduction of explicit EOS definitions and the recommendation for serial physical exams and vital sign monitoring for asymptomatic neonates born to mothers with EOS risk factors.

## Figures and Tables

**Table 1:** Demographic Data of Enrolled Neonates with GA ≥ 35 Weeks.

Characteristics	Before AAP 2019 (*n* = 1639)	After AAP 2019 (*n* = 1401)	*p* †
GA, weeks, mean ± SD	38.3 ± 1.2	38.2 ± 1.2	0.109
Mode of delivery, *n* (%)			0.904
Vaginal delivery	812 (49.5)	691 (49.3)	
Cesarean section	827 (50.5)	710 (50.7)	
Male, *n* (%)	817 (49.8)	740 (52.8)	0.102
Birth body weight, grams, mean ± SD	3072.1 ± 413.9	3080.5 ± 418.9	0.577
Maternal risk factors, *n* (%)			
Preterm labor pain/ROM	68 (4.1)	72 (5.1)	0.194
Prolong ROM	21 (1.3)	10 (0.7)	0.121
Suspected Triple I/Chorioamnionitis	2 (0.1)	2 (0.1)	0.875
Previous infant with invasive GBS	0 (0)	1 (0.1)	0.279
Maternal UTI	0 (0)	1 (0.1)	0.279
GBS in vaginal swab culture			0.210
Negative	18 (1)	23 (1.6)	
Unknown	1621 (99)	1378 (98.4)	
Culture-proven EOS, *n* (%)	1 (0.1)	1 (0.1)	0.912

Note: *n*, frequency; %, percentage; S.D., standard deviation; GA, gestational age; ROM, rupture of membranes; Triple I, intrauterine inflammation, infection, or both; GBS, Group B Streptococcus; UTI, urinary tract infection; EOS, early onset sepsis; AAP 2019, The adapted AAP 2019; *p* †, were calculated using Pearson’s Chi-square test or Likelihood ratio Chi-square test for comparison of proportions among categorical variables between two independent groups, and Independent *t*-test or Wilcoxon rank-sum test (Mann–Whitney U test) for comparison of means between two independent groups.

**Table 2:** Clinical Manifestations of Enrolled Neonates with GA ≥ 35 Weeks and Symptoms.

Clinical Manifestations	Before AAP 2019 (*n* = 291)	After AAP 2019 (*n* = 210)	*p* †
Respiratory distress, *n* (%)	259 (15.8)	191 (13.6)	0.093
Feeding intolerance, *n* (%)	13 (0.8)	9 (0.6)	0.625
Hypotension, *n* (%)	11 (0.7)	10 (0.7)	0.887
Temperature instability, *n* (%)	7 (0.4)	2 (0.1)	0.150
Birth asphyxia, *n* (%)	2 (0.1)	0 (0)	0.191

Note: *n*, frequency; %, percentage; AAP 2019, The adapted AAP 2019; *p* †, were calculated using Pearson chi-square test or Likelihood ratio chi-square test for comparison of proportions among categorical variables from two independent groups.

**Table 3:** Clinical Outcomes After Implementing the Adapted AAP 2019 guidelines for Enrolled Neonates ≥ 35 Weeks.

Outcomes	Before AAP 2019 (*n* = 1639)	After AAP 2019 (*n* = 1401)	*p* †	Odds Ratios * (95% CI)
Investigation, *n* (%)	41 (2.5)	1 (0.1)	<0.001	35.92 (4.93 to 261.47)
Investigation and Antibiotic use, *n* (%)	181 (11)	110 (7.9)	<0.001	1.46 (1.14 to 1.87)

Notes: *n*, frequency; %, percentage; AAP 2019, adapted AAP 2019 guideline; † *p*-values were calculated using Pearson’s Chi-square test or Likelihood ratio Chi-square test for comparison of proportions between two independent groups; 95% CI, 95% confidence interval; * Odds ratios represent the relative odds of the binary outcome occurring in the before AAP 2019 group compared to the After AAP 2019 group.

## Data Availability

The original contributions presented in this study are included in the article. Further inquiries can be directed to the corresponding author.
